# Computational Approaches for Revealing the Structure of Membrane Transporters: Case Study on Bilitranslocase

**DOI:** 10.1016/j.csbj.2017.01.008

**Published:** 2017-01-31

**Authors:** Katja Venko, A. Roy Choudhury, Marjana Novič

**Affiliations:** Department of Cheminformatics, National Institute of Chemistry, Ljubljana, Slovenia

**Keywords:** BTL, bilitranslocase, TM, transmembrane, Membrane proteins, Bilitranslocase, 3D protein structure, Transmembrane region predictors, Helix–helix interactions

## Abstract

The structural and functional details of transmembrane proteins are vastly underexplored, mostly due to experimental difficulties regarding their solubility and stability. Currently, the majority of transmembrane protein structures are still unknown and this present a huge experimental and computational challenge. Nowadays, thanks to X-ray crystallography or NMR spectroscopy over 3000 structures of membrane proteins have been solved, among them only a few hundred unique ones. Due to the vast biological and pharmaceutical interest in the elucidation of the structure and the functional mechanisms of transmembrane proteins, several computational methods have been developed to overcome the experimental gap. If combined with experimental data the computational information enables rapid, low cost and successful predictions of the molecular structure of unsolved proteins. The reliability of the predictions depends on the availability and accuracy of experimental data associated with structural information. In this review, the following methods are proposed for *in silico* structure elucidation: sequence-dependent predictions of transmembrane regions, predictions of transmembrane helix–helix interactions, helix arrangements in membrane models, and testing their stability with molecular dynamics simulations. We also demonstrate the usage of the computational methods listed above by proposing a model for the molecular structure of the transmembrane protein bilitranslocase. Bilitranslocase is bilirubin membrane transporter, which shares similar tissue distribution and functional properties with some of the members of the Organic Anion Transporter family and is the only member classified in the Bilirubin Transporter Family. Regarding its unique properties, bilitranslocase is a potentially interesting drug target.

## 1 Introduction

Transmembrane proteins play a crucial role in maintenance of normal cell functioning; acting as transporters and receptors, playing important roles in signaling pathways, the immune system, and energy production in form of ATP [1]. Therefore, they are of great interest as targets in drug designing, especially for having a double role in pharmacology: (i) indirect by influencing the absorption, distribution, metabolism and excretion (ADME) of drugs, (ii) direct as targets in the inhibition/overexpression of its primary function (the transport of molecules, switching on/off receptors). The membrane proteins are estimated to represent around a fourth of all genes and more than half of market drugs have a mode of action that targets membrane proteins [2]. Knowing the structures and functional mechanisms of more transmembrane proteins could significantly influence drug development and hopefully lead to better healthcare solutions.

Although, the first 3D atomic structure of a membrane protein was experimentally solved in 1984 [3], the majority of membrane protein structures are currently unknown and thus present a major experimental and computational challenge. In the last two years, statistics have shown an impressive increase in solved membrane protein structures by X-ray crystallography or NMR spectroscopy, but the number is still low in comparison with the soluble proteins (~ 380 unique structures, < 1% of all known protein structures in PDB) [4]. The transmembrane proteins are polytopic integral membrane proteins with one or more chains that span the entire biological membrane. The major bottleneck in resolving the tertiary/quaternary structures of transmembrane proteins is the production of suitable stable crystals for X-ray diffraction studies. In this regard, experts in protein crystallography are faced with several technical difficulties in the synthesis, solubilization and maintenance of a functional and stable form of the protein during the crystallization process [5], [6]. Since a considerable amount of effort is invested in breaking these barriers, new methodologies are published on a regular basis. For example, immanent crystallization screens have recently resulted in the development of new scaffold strategies for enhancing the stability of membrane proteins in detergent solutions [7], [8]. Besides application of innovative methodological approaches the rapid emergence of genomics, proteomics and high-throughput technologies (automation, miniaturization, integration, third-generation synchrotrons, electron microscopy) support the enhanced protein structure determination rate [7], [9]. With newly emerging methods, especially in atomic and cryo-electron microscopy, the information gaps in 3D structural datasets (*e.g.* CryoEM map) [10] will hopefully be filled in the near future. At present, the coverage of membrane protein fold space is relatively sparse and, therefore, the use of computational strategies is even more demanding [11].

Providentially, given the current lack of experimental data, numerous highly sophisticated computational approaches are available for the elucidation of protein structures and functions [11], [12], [13], [14], [15]. The computational data-handling combined with existing experimental data inputs enable rapid, low-cost and successful approaches for the prediction of unsolved protein structures. The need for the acquisition of computational models can be directly demonstrated using a group of transporters defined as the solute carrier superfamily. In this superfamily, more than 15,100 human genes are currently annotated as transport proteins, but the X-ray structure only is resolved for 14 of them [4]. In the solute carriers (SLC) superfamily, the family of organic anion transporters (OAT) is the most dominant [16]. OAT members are of interest as potential drug targets due to their uptake of cancer drugs and also as tumor biomarkers [17], [18], [19]. In cases when we have only data on tissue expression and ligand-based experiments without synthesized protein and known gene annotation of protein, computational approaches are the only solutions to overcome the experimental gap. In this context, our work will shed light on the *in silico* research studies devoted to elucidating the three-dimensional (3D) structure of the bilirubin membrane transporter bilitranslocase (BTL, Uniprot O88750), which shares similar tissue distribution and functional properties with some of the members of the OAT family and is the only member classified in the Bilirubin Transporter Family (BRT) in the Transporter Classification Database (TCDB 2.A.65) [20]. As its name determines, it is strongly involved in the bilirubin uptake from the blood into the hepatocytes [21], [22]. However, BTL is not responsible only for the transport of bilirubin into the hepatic cells; the biochemical studies performed so far reveal a much broader tissue expression (parietal cells of the gastric mucosa, proximal renal tubule cells and the vascular endothelium cells [23], [24], [25], [26], [27]), where BTL acts as an efficient influx device for a wide variety of vitally important endogenous substrates and drugs [28], [29], [30]. BTL is known to transport a wide variety of poly-aromatic compounds, but the most unique feature of BTL is the ability to transport nucleotides, which, to the best of our knowledge, no other membrane transporter is able to do [30]. These peculiarities make BTL a hot target for future investigations, especially in the context of endogenous drug delivery systems to intracellular targets. Consequently, there is an urgent need for the elucidation of its 3D molecular structure and the clarification of its substrate specificity, as well as its functional and mechanistic significance. Several experimental studies have already focused on BTL, but its 3D structure and functional mechanism still remain unclear due to problems with its synthesis. BTL is a 38.22 kDa transmembrane protein with 340 amino acids translated from a 1026 bp long mRNA sequence (GenBank: Y12178.1), which was isolated already in 1978 from liver cytoplasm of *Rattus norvegicus* [31]. The BTL gene remains unidentified until now. However, it is of interest that the mRNA of BTL shares 94% homology (RNO30106 coding/coding overlapping transcript in antiCODE database [32]) with antisense mRNA of ceruloplasmin, a plasma glycoprotein that has localized its functional gene on human chromosome 3q21-q24 and pseudogene on chromosome 8q21.13-q23 [27]. In spite of several efforts, the 3D structure of BTL is not yet resolved and no sequence homology with any other known membrane protein has been found [20]. Interestingly, BTL has a conserved bilirubin-binding motif, which plays a central role in ligand binding and is similar to globular alpha-phycocyanins [20], which are ancient biliproteins present in cyanobacteria.

The aim of this review is to demonstrate the usage of various computational methods for the structural determination of transmembrane proteins; examples are given for a case study of bilitranslocase. It needs to be emphasized that the selected case study is of special importance, as it demonstrate how to tackle the challenges of membrane protein structure analysis while lacking a template-based homolog. The structural studies include: sequence-dependent predictions of transmembrane regions, predictions of transmembrane helix–helix interactions, their arrangement in membrane models and testing their stability with molecular dynamics simulations.

## 2 Approaches for Predicting Protein Structures From Amino Acid Sequences

Protein structure prediction tools are nowadays the standard toolkit in life science research and a variety of *in silico* applications are available. The computational programs for protein structure elucidation include various statistical and supervised learning algorithmic techniques [13], [33] for solving the sequence-structure alignment and the identification of structural units: the very regular local sub-structures (α-helixes and β-sheets) in the secondary structure [34] and the protein domains (distinct and compact intra-chain units that could fold independently into other chain parts) in the tertiary structure [35]. All the computational algorithms were developed on the assumption that different proteins fold into similar 3D units because they have similar interaction patterns among their residues and between the residues and the environment [36].

Sequence-based tools for secondary and tertiary protein structure prediction are an important and challenging field of research in structural bioinformatics. Over the last two decades, the methods for the prediction of secondary structural elements in proteins have obviously progressed. Nowadays, secondary structure prediction methods are seldom used alone, but are mostly used to provide constraints for the tertiary structure prediction methods [11]. Consequently, the accurate secondary structure identification, the ever-increasing size of the training sets and the optimized alignment methods have also improved the sensitivity and usability of tertiary structure prediction methods [37]. The most widely used techniques for tertiary structure prediction are homology modeling, fold recognition (protein threading), and *de novo* (*ab initio*) protein structure prediction. The techniques vary in their basic concepts; (i) the first two are based on fitting a protein sequence to a structural template, requiring the identification of one or more homologs that the structure is based on (*e.g.* PROSPECT [38], RAPTOR [39], pGenTHREADER [40], pro-sp3-TASSER [41], MODELER [42], I-TASSER [43], iMembrane [44]), (ii) the third involves the use of simulated annealing algorithms, which are based on the general principles that govern protein structure and energetics and therefore does not require a template (*e.g.* ROSETTA [45], FRAGFOLD [46], PROTINFO [47], FILM [48]). Each software tool has its specificities and advantages, and use different energy functions and computational methods, but protein 3D structures' building methods generally achieve relatively similar levels of accuracy. Especially among template-based methods, accurate predictions were observed when sequence identity was above 30%. Therefore, among the tertiary structure prediction methods, it is interesting to note the really outstanding popularity of homology modeling with MODELER [42]. However, for TM protein prediction Kelm et al. [49] presented the TM-specific homology-based tool MEDELLER and used performance analyses to demonstrate that MEDELLER outperforms the most popular homology modeling tool MODELER. The low usability of *ab initio* techniques is reasonable because they are much more computationally intensive thus limited to smaller proteins and are less accurate than template-based methods. Since template-based methods are currently the most widely used computational approaches for protein structure prediction [11], they are unfortunately still mostly applicable for soluble proteins, for which several crystal structure templates are available, while only few or no homologs of known structures exist for majority of TM protein families. In this regard, *ab initio* methods seem more suitable for membrane protein structure predictions, but due to the size restriction associated with these methods, they are now mostly used for building the non-homologous loop regions only [33]. Some exceptions exist, such as the methods FILM [48] and RosettaMembrane [50], which were specially modified for TM proteins and are capable of predicting TM structures in sizes up to 300 residues. Therefore, in membrane protein structural bioinformatics, the topology prediction methods dominate, as the accuracy of models built using template-based methods is highly restricted by the availability of good structural templates and near-perfect alignments [11]. The structure elucidation of polytopic integral membrane proteins starts with especially sophisticated tools for the topology prediction of TM regions. Several TM-specific predictors are available for this step and will be further compared in this review with a practical representation of use on the BTL transporter. To date, the tertiary structure prediction of membrane proteins remains a significant challenge, but the recent development of methods specifically optimized to align membrane proteins (AlignMe [51], MP-T [52], PRALINE™ [53], TM-Coffee [54]) enable promising progress. Significant advances have been made in the last decade on methods for predicting helix–helix interactions, but the methods for the optimal packing of β-sheets are still in the development phase due to the severe shortage of known structures and higher diversity in topology [12].

However, among membrane protein families, some successful cases of the use of template-based and *ab initio* methods have also been presented [49], [50], [55], [56]. G protein-coupled receptors (GPCRs) [56] and SLC transporters [37], [55] are widely studied and already structurally resolved membrane proteins, therefore members of these groups can now be modeled with an accuracy sufficient for virtual screening. While, there is concern about the reliabilities of the structural models of membrane proteins that were attempted using methods that are sophisticated for soluble proteins [49]. The major concerns and drawbacks of such models are: (i) the models were built from template structures with low sequence identity to the target sequence, (ii) the alignment methods were based on statistical potentials derived from soluble proteins and did not take into account the fact that membrane proteins have different amino acid substitution preferences from their soluble counterparts, (iii) unique properties like specific interhelical interactions were not considered [11]. Recent studies demonstrate that if such preferences were included, the alignments were improved and consequently the membrane protein models were also improved [52], [55].

### 2.1 Sequence Dependent Predictions of Transmembrane Regions

The first step in transmembrane protein structure prediction is the identification of the α- or β-TM regions. TM regions are, by concept, all the peptide chains within the nonpolar region of the lipid bilayer. The TM regions have in common the organization of chains into a plethora of predominantly hydrophobic residues that are energetically suitable for the hydrophobic membrane environment and have aromatic/charged residues at the terminal positions, which are more suitable for the membrane-water interface. The predictions of the TM regions are based on inputs of amino acid sequences and can in general be approached from two distinct perspectives: (i) pattern-based (hydrophobic or sequence pattern classification), (ii) homology-based (making comparison to existing data of homologs). In the first concept, the structural features are predicted based on algorithms using hydrophobicity scales or sequence similarity [31], therefore applicability is theoretically equal for homologs and non-homologs. The second concept is based on algorithms, which besides sequence patterns also include evolutionary information, so the probability of prediction is dependent on or biased with the homology rate [13], [33], [57]. Interestingly, all integral membrane proteins with currently known high-resolution structures are strictly homomers and not mix assemblies of both TM structural units. Therefore, membrane proteins are classified in two highly distinct structural classes: bundles of α-helices or β-barrels [58]. In this regard, the predictors for each class were diverged and developed separately to achieve better precision in the predictions of the TM structural units.

Both types of predictors apply various methods based on different algorithmic techniques, which can in general be categorized into three classes: physicochemical methods, statistical methods and machine learning methods [13], [33], [59]. Several physicochemical methods based on hydrophobicity indexes [60], [61], [62] and several hydropathy analyses that identify long stretches of hydrophobic residues are available for the topology prediction of TM helices (KD [63], PRED-TMR [64], SOSUI [65], TM Finder [66], TopPred2 [67]) or barrels (BBF [68], BOMP [69]). These methods are successful at identifying the hydrophobic core of TM regions, but cannot precisely determine the ends of TM residues, therefore they are commonly corrected with the cutoff values for membrane-spanning residues (values calibrated against known structures of membrane proteins) [70]. The propensity-based methods rely on statistical analysis of the occurrence of certain residues in the secondary structures of known protein structures (*e.g.* for helices (MEMSAT3 [71], SPLIT [72], TMpred [73]) and for barrels (Freeman–Wimley approach [74])). The applicability of these methods is mostly tempered due to the limited number of known atomic resolution structures of transmembrane proteins. The machine learning methods are rapidly evolving and are based on learning algorithms like Support Vector Machines, Hidden Markov Models and Neural Networks. Regarding various performance analyses, these methods are recognized as the most advanced and accurate [57], [59], [75]. Some of them are able to predict at least three structural states: cytoplasmic region, TM region and extracellular region. The basic principle of these methods is to train based on a set of input/output pairs and to detect correlations that facilitate pattern recognition and then evaluate the prediction probabilities (*e.g.* for helices HMMTOP [76], ENSEMBLE [77], Philius [78], TMHMM [79], MEMSAT-SVM [59], TOPCONS [80], PredαTM [81] and for barrels B2TMPRED [82], ConBBPRED [75], PRED-TMBB [83], TMBpro [84], PredβTM [57], TMBHMM [85] *etc.*). Machine learning approaches are best for solving problems in the absence of general theories when there is a large amount of data with noisy patterns and are thus ideal for use in the explication of protein complexity.

Due to the shortage of the high-resolution TM protein structures, current computational methodologies probably do not yet cover all the properties of the TM domains in the proteome. Therefore, currently available algorithms are based on datasets that do not completely sample the TM protein data space of living organisms. The lack of information is especially substantial in the case of β-TM proteins. Nevertheless, the current predictors in most cases seems robust and relatively accurate, but the performances are significantly overestimated [57], [59]. Consequently, the tendency in structural bioinformatics is to develop algorithms independent of explicitly resolved evolutionary information, which are potentially capable of giving accurate TM region predictions for the novel non-homologous membrane protein sequences. Even more, besides identifying the TM regions, the interest in using predictors for genome annotation is also increasing [57], [59], [86]. In spite of the abundance of predictors, the researcher's intent is always to use the most up to date methods based on all the currently available sequence and structural data. Thus prediction methods are gradually evolving by including the new protein structure information that becomes available [87]. However, with new experimental strategies, the availability of high-resolution structures is rapidly increasing and, consequently, coverage of the complete transmembrane protein space is approaching.

#### 2.1.1 Accuracy Measurements and Performance Analyses of Secondary Structure Predictors

Comparing the accuracy of different structure prediction methods is not a univocal task due to a number of methodological challenges, such as the use of different protein data sets or homologous proteins, various secondary structure definitions, and various accuracy measures. For TM proteins in particular, these problems are of special relevance according to a relatively limited high-resolution structure database, which often leads to the overestimation of prediction accuracy [33]. Thus the best strategy to improve prediction accuracy is to make a consensus prediction based on compiling the prediction results from different methods [75], [81].

In general, measurements of the accuracy of predictors are based on a comparison of the predicted and experimentally observed TM segments. Most of the measures are defined for a two-state classification scheme (TM segment or not TM segment), which are further differentiated based on per-residue and per-segment accuracy. Accuracy measurements are evaluated using confusion matrix data, which include the numbers of correct/incorrect predictions (true/false positive, true/false negative) and are defined as accuracy, sensitivity, specificity, and the Matthews correlation coefficient. Per-residue accuracy involves evaluating the accuracy of prediction for particular residues (the percentage of residues predicted correctly in TM regions). Per-segment accuracy is more commonly used and based on a comparison of predicted *versus* experimentally observed TM segments in a data set and are defined as per-segment sensitivity, positive predictive value and segment overlap [88], [89]. The sensitivity is the percentage calculated from the number of correctly predicted TM regions divided by the number of experimentally observed TM regions in the data set. The precision is specified as a percentage of all the TM regions that are correctly predicted. The segment overlap (SOV) is a measure of the similarity of the predicted and experimentally observed TM segments [88].

In the case of α-TM region predictors, several performance analyses have been implemented [57], [89], [90], [91], [87]. All have in common that the simple predictors based on hydrophobicity scales perform fairly well for the prediction of α-TM helices, but are less accurate than the algorithms based on amino acid distribution preference, sequence alignments and evolutionary information. Overall, hydrophobicity-based methods overestimate the TM helices, mostly because of the inability to distinguish TM helices from signal peptides and hydrophobic regions outside of membranes. To avoid these problems, some authors propose firstly to use the prediction methods that are effective in identifying signal peptides as a pre-filter [92], prior to analyzing with TM topology predictors. Furthermore, some α-TM region predictors already contain specialized sub-models for the recognition of signal peptides [78], [59], [87], [93]. The studies have shown that advanced machine learning methods with complex algorithms using multiple sequence alignments outperform hydrophobicity-based predictors, but have a tendency to underestimate the TM helices [59]. Based on a benchmark analysis of ten machine learning methods by Nugent and Jones [59], the MEMSAT-SVM method was demonstrated as the most accurate one. In another benchmark analysis, Tsirigos et al. [87] reported that the updated version of TOPCONS, a consensus method that combines several prediction methods, offers state-of-the-art performance. On the other hand, an outstanding methodological exception is the PredαTM algorithm based on an effective mathematical representation of a peptide sequence. In this algorithm, the amino acid composition of TM regions and sequence patterns are characterized by mathematical descriptors derived from the amino acid adjacency matrix [81], [91], [94]. The recent performance analysis of 17 state-of-art α-TM region predictors carried out on 38 benchmark sequences including both single-spanning and multi-spanning membrane proteins from both prokaryotes and eukaryotes is presented in Table 1. It is clear that some of the advanced methods perform better than others. Those achieving the highest sensitivity and precision are PredαTM [81], [91], TOPCONS [80], HMMTOP [76], Philius [78], OCTOPUS [95], SCAMPI [96] and SVMtop [97]. The PredαTM predictor [,91] is independent of any explicitly expressed evolution information, thus has an added advantage over the aforementioned algorithms when predicting TM helices for proteins that show very low or no sequence homology and it could also be useful in the context of entire proteomes analysis. Nugent and Jones [59] also demonstrated on a number of complete genomes that the MEMSAT-SVM method can effectively discriminate between TM and globular proteins and thus could be suited to the whole genome annotation of α-helical TM proteins. Furthermore, Tsirigos et al. [86], [87] examined the ability of prediction methods to distinguish between membrane proteins and soluble ones, and obtained the best performances with the methods utilizing multiple sequence alignments like TOPCONS [87] and Phobius [93]. The low agreement between predictions obtained using different methods shows that the global properties of the membrane proteome are still a hot matter of concern [86]. Therefore, for a relevant differentiation between globular and membrane proteins, it is suggested to apply a consensus approach based on the analysis of results from various currently available predictors.

The old performance analyses for the prediction of β-TM regions [75], [98] showed that methods based on the Hidden Markov Model like ProfTMB [80], HMM-B2TMR [81] and PRED-TMBB [68] were the best in terms of SOV, per-residue and per-segment accuracy. Furthermore, Bagos et al. [75] demonstrated that the consensus prediction method ConBBPred performed significantly better than each individual available predictor. On the other hand, Freeman and Wimeley [74] showed that a good statistical approach can surpass the accuracy of machine learning methods. However, due to the amphipathic nature of the β-TM regions, the hydrophobicity was recognized as an inefficient discriminating factor and prediction accuracy was improved with the incorporation of non-linear statistics and evolutionary profiles. For most algorithms, the accuracy was overestimated regarding a limited benchmark set and the probable inclusion of homolog proteins in the original models dataset. The newest benchmarking analysis for β-TM predictors presented in Table 1 shows that the machine-learning-based PredβTM predictor currently outperforms all the state-of-art β-TM region prediction methods [57]. Although, the consensus method ConBBPred, which makes predictions by combining outputs from several algorithms (PRED-TMBB [83], ProfTMB [99], HMM-B2TMR [100], *etc.*) achieves a considerably high degree of precision, it radically fails in sensitivity, being the lowest among all the compared methods. In general, the β-TM proteins have little sequence identity, even if only the TM regions are considered. Therefore, the PredβTM algorithm, which is independent of any explicitly given evolutionary information [43], has an important advantage over most of the methods based on sequence profile data like B2TMpred [82], TBBpred [101], TMBpro [74] and TMBETA-NET [102]. Moreover, besides concerning the identification of TM proteins, it can also be useful for gene annotation in gram-negative bacterial genomes [57], [74]. We can emphasize that the prediction of β-TM regions still remains a difficult problem. The majority of predictors have limited prediction accuracy due to the limited availability of non-homologous β-TM protein structures [4] and the probability of the existence of β-TM protein families that have not yet been described.

#### 2.1.2 Case Study: Step 1 – BTL TM Regions Prediction

**Fig. 1:**
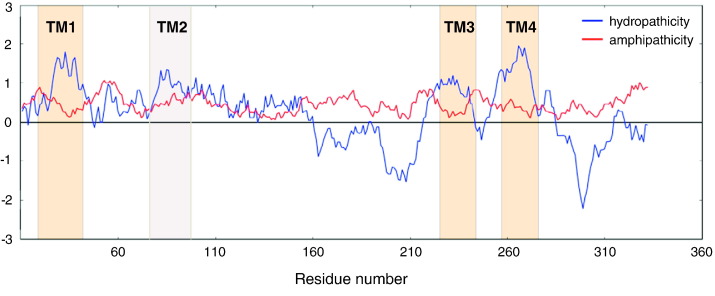
The prediction of the bilitranslocase transmembrane regions. Hydropathy analyses of the amino acid sequence using the Kyte-Doolittle scale (WHAT 2.0, [63]): the blue line represent hydropathicity and the red line represent amphipathicity. The columns represent the position of the predicted transmembrane regions (helices): *orange* – regions predicted using HMMTOP, PredαTM, SCAMPI, TMpred, TopPredII, TOPCONS; gray – extra region predicted using PredαTM, TMpred, TopPredII, Philius, MemBrain, PRED-TMR [81], [91]. The transmembrane regions TM1, TM3 and TM4 are consistent with hydrophilic peaks, while the detection of TM2 is indefinite due to the ambiguous hydropathy profile. (For interpretation of the references to color in this figure legend, the reader is referred to the web version of this article.)

BTL is a 340 amino acids long TM protein of an uncharacterized gene and unknown 3D structure. Knowing only the mRNA amino acid sequence, Roy Choudhury et al. [81], [91] made a prediction of the BTL TM regions by testing 17 freely available α-TM region predictors. The analysis of the predictions resulted in the identification of four TM helices: TM1 (24–45), TM2 (73–95), TM3 (221–238) and TM4 (258–277). Only three algorithms (PredαTM, TMpred and TopPred II) predict all four TM regions. The algorithms PRED-TMR, MemBrain and Philius predict the transmembrane regions TM1, TM2 and TM4. On the other hand, the predictors HMMTOP, SCAMPI and TOPCONS predict the transmembrane regions TM1, TM3 and TM4. The rest of the algorithms only predicted the regions TM1 and TM4. Interestingly, the SOUSI predictor classifies BTL as a globular protein. The hydropathy sequence profile shown in Fig. 1 can give an explanation why all the predictors reliably identified the TM1 and TM4 regions, while the identification of TM2 and TM3 regions was more ambiguous. Furthermore, based on these computational analyses of BTL secondary structure units, experiments were designed and the ambiguous TM2 and TM3 helices were successfully confirmed using multidimensional NMR spectroscopy [104], [105], [106].

## 3 Determining the Structure of Assembly of the TM Regions

For the elucidation of the membrane protein structure and functional mechanism, knowing the exact assembly and arrangement of its structural domains is crucial. The interactions between TM regions play a critical role in the function, assembly and oligomerization of membrane proteins. In these interactions, physical forces (*e.g.* polar and aromatic interactions, salt bridges) among two or more helices or strands are included [12], [14]. The determination of the optimal packing of the TM regions includes effective sampling strategy and the usage of complex optimization algorithms following the principles from the laws of quantum mechanics. Theoretically, the optimal arrangements have the minimal free energy geometry [13]. In general, the simplified two-stage protein folding model of membrane proteins enhances the solving of this complex folding problem by including the results from TM regions predictors, which provide reliable information on the assembly of secondary structure units. Further, with mathematical operations, the minimal inter-unit interaction energy is calculated based on scoring functions that represent the molecular determinants of helix–helix/strand-strand interactions [12], [14], [111], [112]. The representation of such determinants is the most challenging part and over the years, significant progress has been made in the field of tertiary structure prediction for helical TM proteins [113], [114], [115], while for β-TM proteins the identification of molecular determinants is moderate [33], [85], [116]. While the folding of globular proteins can be predicted using methods like homology modeling and threading, such methods should be applied very carefully and critically for the prediction of membrane protein folding, due to the shortage of atomic resolution TM protein structures. The accuracy of prediction is questionable if specific membrane preferences are not included in the protocol [115], [37], [49]. Once the optimal packing is determined, the stability of the predicted arrangement in the lipid environment could be further studied by using molecular dynamics (MD) simulations [117]. The MD simulations can be computationally expensive and thus the size of the simulated system should be reasonably limited or a multiscale approach used [117], [118]. Regarding the case study protein, the details of practical examples of analyses of the arrangement of the four helices of bilitranslocase is presented.

### 3.1 Predicting the TM Helix–Helix Interactions

So far, the known membrane proteins (ion channels, transporters and receptors) in living organisms are mostly built of helices [119], therefore substantial research is done on studying helix–helix interactions [12], [14], [114], [120] and less is known about strand-strand interactions. The correct folding of polytopic membrane proteins firstly involves individual helix–helix interactions and then multiple helix-dimer interactions need to be controlled and aligned to result in the final higher-ordered oligomeric structure. In contrast to soluble proteins, our knowledge of the factors that control the oligomerization of membrane helices is limited. Taking into account that the membrane environment has unique chemical and physical properties, the rules applicable for interactions between soluble segments are not necessarily valid within the membrane [121]. Of interest is the recent extensive research by Zhang et al. [115] on helix–helix interactome, which provides the first comparison of TM and water-soluble helical pairs. Although both types of proteins have a similar pair geometry, the interactions are different. The involvement of larger charged residues and more interhelical hydrogen bonds are observed in water-soluble structures [115]. Among the best known TM helix dimerization factors are the conserved motifs GxxxG, QxxS and WxxW [14], [114], [120], interhelical residue pairs/triplets [122], polar clamp, and serine/leucine/glycine zippers [12], [13], [14]. Although it was believed that TM helix–helix interactions are mostly dominated by the rule of amino acid sequence motifs, experimental evidence suggests that interactions between TM helices are much more complex, resulting in an aromatic pattern (aromatic-XX-aromatic) [123] or interactions between TM sequences that do not contain any recognizable motifs [14]. Cymer et al. [114] highlighted the sequence context in their study, as well as lipid bilayer properties that highly modulate the structure and stability of a helix dimer. Recent studies have also revealed the particular physical properties of lipids or membranes that play roles in the protein folding process [124]. Most of the studies evaluate the helix–helix interactions by molecular dynamics (MD) simulations [12], but also several *in silico* methods exist for the prediction of pairwise helix–helix interactions from the primary sequence. In general, they are generated using various machine-learning-based algorithms involving either residue contacts (per-residue) or complete TM regions (per-segment) (*e.g.* MemBrain [125], MEMPACK [126, PROFcon [127], SVMcon [128], TMHcon [112], TMhit [113], TMhhcp [130], IMP [131]). However, the tools available for the prediction of helix–helix interactions in globular proteins [127], [128] perform relatively poorly in TM proteins. The reason is probably the differences between TM and globular interaction motifs [14], [114], [115], [121]. For example, the helix–helix interaction prediction performance for five predictors (the TM protein contact predictors MEMPACK, TMHcon and TMhit, and the globular protein contact predictors PROFcon and SVMcon) was assessed based on a data set of 74 sequences, which contained at least two TM helices [106]. The TM proteins' specific predictors TMhit and MEMPACK achieved the highest accuracy (> 60%). These predictors are not solely able to predict residue contacts and helix–helix interactions, but can also present a modest estimation of the lipid exposure and helical packing arrangement of TM proteins [129]. For example, studies on archaerhodopsin [126] and cytochrome C oxidase [129] revealed a high level of consensus among the predicted interactions and the observed helical packing arrangements derived from the crystal structure. Since such predictors are only capable of constructing packing arrangements of proteins with up to 13 TM helices [129], more advanced tools for predicting the assemblies of proteins were developed, which are further described in Section 3.2.

#### 3.1.1 Case Study: Step 2 – the Prediction of Transmembrane Helix–Helix Interactions in BTL

**Fig. 2:**
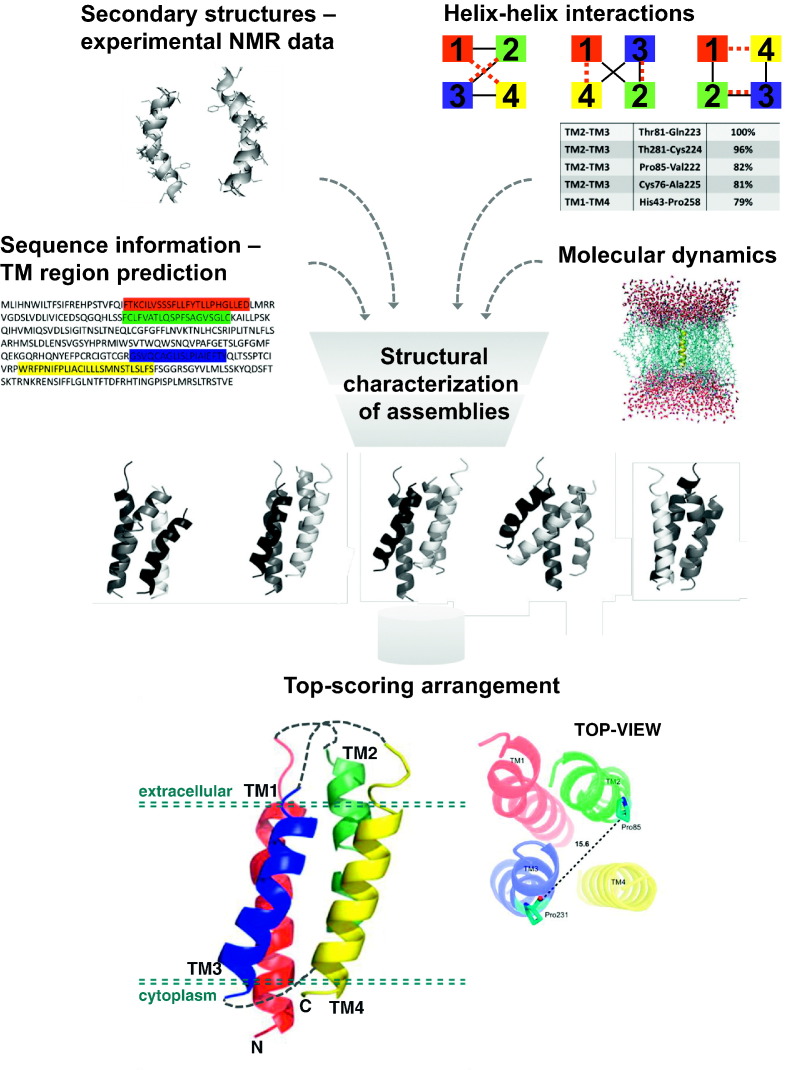
The structural characterization of bilitranslocase, a membrane protein with no structural homolog. A hybrid approach that integrates data from diverse computational and experimental methods [91], [104], [105], [106] was used for determining the structure of the transmembrane assemblies. At the bottom, the best scoring arrangement of the four transmembrane helices of bilitranslocase (Top2 [106]) is presented; TM1 (24–45), TM2 (73–95), TM3 (221–238) and TM4 (258–277).

The Integrative Modeling Platform (IMP) [131] was used to predict the per-segment helix–helix interactions in BTL. The sequences of TM regions with defined topologies predicted by the TM region predictors served as the input. The rigid body representation for each TM region was built based on Discrete Optimized Protein Energy (DOPE) [132] using specified statistical potentials for TM proteins from SaliLab and without considering the experimental NMR or MD data that are available. All possible TM helix pairs were considered to predict the probable interactions, because TM helices can interact in multiple ways [122]. Each member of a helix–helix pair was allowed to access all the permissible combinations of rotational, translational and tilting movements. The permitted range of tilting angles, diameter, excluded volume and packing restraints for each TM helix pair were based on the Orientations of Proteins in Membranes (OPM) database [133], which lists the spatial arrangements of alpha TM proteins with respect to the hydrocarbon core of the lipid bilayer. The stability of the possible transmembrane helix–helix interactions were optimized using the scoring function based on interaction data of TM helices from the OPM database. It was assumed that all the TM helices could interact with each other; therefore each helix–helix pair interaction was optimized and scored independently. The highest scoring transmembrane pairs with scores above a predefined threshold were considered to be interacting. The four BTL predicted TM regions give rise to six possible helix–helix interaction pairs. Among them, the pairs TM2-TM3 and TM1-TM4 have the lowest energy scores and therefore showed the most optimized configurations and were reported to be interacting (Fig. 2) [106]. The TMhit algorithm [113], which predicts per-residue helix–helix interactions, was also applied. The TM2-TM3 and TM1-TM4 helix–helix pairs were found to interact, and these are the same pairs as those previously predicted by IMP. The existence of the TM2-TM3 pair in the SDS micellar environment was also independently supported experimentally by NMR spectroscopy and FRET efficiency [106].

### 3.2 The Prediction of TM Assemblies

To elucidate protein functions the models of high order assemblies are essential. Using current computational models, the determination of the positions and orientations of all components can be unambiguous, yet continuous improvements using the implementation of new scoring functions are in progress [10], [12], [125]. However, such models are hopefully valuable for directing experimental studies on TM proteins where structural data is currently unavailable. Various prediction methods that generate the pseudo-atomic models of the assembly are available (*e.g.* MultiFit [10], RosettaMembrane [134], FRAGFOLD [46], FILM3 [135], IMP [131]). Nowadays they are gradually increasing in number due to the rise in availability of datasets from atomic- and low-resolution (*e.g.* cryo-electron microscopy (cryo-EM) density maps, small-angle-X-ray scattering (SAXS) profiles), genomic and proteomic techniques. In general, the use of such tools is less user-friendly due to the complex computational protocols and is not so appropriate for beginners. For an accurate and careful performance, the knowledge of an expert in structural bioinformatics is indispensably needed.

The methods for predicting high order arrangements of TM regions are based on the constraints of the interacting TM helix–helix pairs and moderate helical packing arrangements identified in advance using the methods described in the Section 3.1. This information is especially valuable for *ab initio* modeling methods [46], [134], [135], as it reduces the conformational search space and computational requirements [129]. In general, the probable arrangements of the predicted TM helices are determined with the identification of the most populated and energetically favorable arrangement type. The arrangement with the most favorable energy can be identified with an effective sampling strategy defined in an optimization algorithm. Probably the most popular optimization algorithm is based on Monte Carlo simulations, which can perform an optimized sampling of the protein search space [131]. The algorithms include scoring functions, which are composed of restraints and evaluate how well a model agrees with the information from which the restraint was derived. In most cases the restraints encode both what is generally known about protein structures and what is known about a particular structure of interest. Thus, a candidate model with the highest score is consistent with all the input information. Normally, by increasing the amount and quality of information included in restrains, the precision and accuracy of the resulting model increases [15].

In recent years, researchers realized that the detailed structural characterization of complex proteins or macromolecular assemblies is practically impossible to achieve using any single experimental or computational method. Therefore, scientists were and are still searching for a way to overcome this barrier. Until now, hybrid approaches that integrate data from diverse experiments (*e.g.* X-ray crystallography, cryo-EM, immuno-electron microscopy, NMR and FRET spectroscopy, SAXS, immunoprecipitation, genetic and proteomic interactions) are highly efficient and prospective [15]. For example, integrated approaches like MultiFit [10] and IMP [131] can contribute to the structural characterization of biomolecules ranging in size and complexity from small peptides to large macromolecular assemblies, by integrating data from diverse experiments. MultiFit [10] is based on a protocol for the simultaneous fitting of high-resolution structures of components into a density map of the whole assembly. On the other hand, IMP [131] is even more advanced and has scoring functions suitable for integrating various types of experimentally obtained data like SAXS profiles, NMR spectroscopy, cryoEM and imunoEM images and density maps, proteomics data, fingerprints or affinity purification. Numerous complex protein structures have been successfully solved using this platform. The 26S proteasome structure [136] was determined with the integration of data from an electron microscopy map, proteomics data, and comparative protein structure models. The structure of the bacterial type II pilus was identified from NMR data and X-ray crystallographic structures of constituent proteins [137]. The value of IMP especially, is demonstrated by solving the human RNA Polymerase II [138] and yeast nuclear pore complex [139] with integrating information from multiple experimental and computational sources.

#### 3.2.1 Case Study: Step 3 – the Prediction of BTL TM Regions Arrangement

In the case of predicting the arrangement of the TM regions of BTL, the input parameters were the TM region sequences, topology, loop connectivity, conformations from MD simulations [104], [105], the predicted interactions in the pairs TM2-TM3 and TM1-TM4 [106], and the filter parameters [121]. The discrete conformation space was defined with restrictions, such as the transport channel diameter and the tilt and depth of the TM helices [122], [133]. The chosen value for the transport channel diameter is dependent on the number of TM regions present in BTL [133]. So defined initial configurations, were used as constraints in the Monte Carlo simulations coupled with the DOMINO algorithm in the MultFit toolkit [10]. The DOMINO algorithm applied an approach to efficiently discover global optimal solutions within the discrete sampling space [10]. The resulting subset solutions were then combined to obtain the global solution. The distribution analysis illustrates that even though the highest scoring structure favors the TM1-TM4-TM3-TM2 arrangement, the most frequently observed was the TM1-TM2-TM4-TM3 arrangement type (shown in Fig. 2), which has the predicted interacting TM helix pairs positioned diagonally opposite each other [106].

### 3.3 Stability Predictions of TM Assemblies with Molecular Dynamics (MD) Simulations

MD simulations can be used for stability predictions concerning the TM regions in a lipid bilayer and even more, the internal motions and conformational changes of the TM protein domains can be assessed [118]. MD simulations have ever-increasing potential in the elucidation of all biological dynamic systems by generating dynamic models that illustrate contact interactions and internal motions that are playing a functional role. In contrast to crystal structures, membrane proteins in their native membrane environments are not static, but rather have a variety of conformations [140]. Therefore, MD simulations allow the *in silico* reconstitution of structures and their movements in a bilayer environment. Bondar et al. [141] reported that the membrane protein conformational dynamics is associated with changes in the interhelical hydrogen bonding. As hydrogen bond interactions can be extracted from MD simulations, running MD simulations is crucial for understanding the H-bond dynamics and the stability of protein domains. Various computational dynamics methods can be used to investigate and study the dynamics of the biomolecular system [142]. Since membrane systems are large in size, the atomistic simulations are generally time-consuming, therefore the introduction of coarse-grained models in MD simulations speeds up this process [117], [143]. In general, methodologies are highly dependent upon setting the role of the solvent in protein dynamics and the availability of a suitable potential-energy function to describe the energy landscape of the system with respect to the relative coordinates of the constituent atoms, which represent the structure of the studied system [117]. In this regard, the Lipidbook, a public database for force-field parameters used in simulating biological membrane systems [144], and the Course Grained Database (CGDB) [143] can be useful. Significant progress has been made in the performance of membrane protein MD simulations in recent years. For example, important functional implications of TM protein interactions were discovered in the KscA potassium channel [145], the MscL mehanosensitive channel [146], the sugar transporter LacY [117], aquaporin [140], *etc*. [12]. In this way, specific residues that play important structural and functional roles or influence selectivity have been characterized [13], [118], [147], [148]. Moreover, since a key element in the refinement of 3D protein structure prediction is to incorporate reliable structural information into the protocol, it is essential that various prediction tools that enable the inclusion of MD data in the computation of 3D structural models already exist [149], [150], [131]. Moreover, to make MD simulations in bilayers easily available to interested stakeholders, the MemProtMD software was recently developed. This is a completely automated high-throughput computational pipeline for the insertion of membrane protein structures into an atomic resolution model of the membrane environment, and the results of simulations are publicly accessible in the MemProtMD database [151].

#### 3.3.1 Case Study: Step 4 – MD Simulations of BTL TM Regions

The preparation of the system of BTL TM helices for MD simulation was done using CHARMM, one of the popular molecular modeling programs [152]. The lipid system was based on the standard dipalmitoylphosphatidylcholine (DPPC); additional layers of water molecules were added above and below the lipid bilayer to enable full system solvation. The Monte Carlo simulations are commonly used for generating a set of representative configurations under given thermodynamic conditions such as temperature and volume [153]. Thus, they were also performed in this study. Numerous 20 ns trajectories were obtained using different force constraints; all the trajectories were accurately analyzed to get the overall view. The stability of the TM regions was confirmed, if the RMSD values and backbone torsion angles were stable. In BTL, during the 20 ns long MD simulations, an overall stable RMSD was observed for each helix. The analysis of the torsion angles confirmed that the α-helical secondary structure of the TM2 and TM3 regions remained preserved in the membrane environment [104], [105]. In addition, the formation of Proline-induced kinks was observed in TM2-TM3 [106] and the authors suppose that this is crucial for the conformational change during BTL substrate transport. Kinks are well known to have a functional role in membrane proteins [147] and the detailed research on TM helices showed that majority of kinks are associated with Pro residue [148].

## 4 Summary and Outlook

The interdisciplinary approach is essential in complex bio-molecular studies, where experimentalists collaborate with computational chemists, who analyze large quantities of existing data and may quickly provide structural and functional information that is fundamental to guiding the further directions of investigation. Since the 3D structure of transmembrane proteins in general are mostly unknown due to technical difficulties in experimental sample preparations, computational approaches are the only way to elucidate the potential structural features of these proteins. This is a very challenging task due to the gap in the known atomic structures, which is especially notable in TM proteins. Although a variety of computational methods for the *in silico* determination of 3D protein structures are available, not all of them are sufficiently applicable for membrane proteins. The methods that are really sophisticated for membrane protein structure predictions are currently limited, but this field of research is of high interest and existing methods are constantly being updated or new ones developed with integrated modifications for TM proteins. Currently, the use of various template-based methods like comparative modeling and threading was able and surprisingly efficient for membrane proteins that share sequence homology with structurally resolved proteins. But what can be done in most of the cases when no structural membrane template exist that can be reliably aligned to the selected target? The use of *ab initio* methods is one of the possible choices, but is nowadays still limited to small protein systems. In contrast, an interesting hybrid approach, primarily used for the structural determination of macromolecular assemblies, is available for use in the structural characterization of membrane proteins. This approach was demonstrated by a case of structural elucidation of bilitranslocase, a transmembrane protein with a known amino acid sequence but with no available information about its 3D structure or structural homolog. The integration of diverse existing data obtained from computational and experimental methods was the only way to make further progress in the elucidation of its structure. Such data integration with its iterative series of satisfaction to the input spatial restrains resulted in the 3D computational model of TM region arrangement of BTL, which represents an optimal arrangement of four membrane α-helices. The methodology based on integrative approaches can maximize the accuracy and efficiency of structural characterization using constraints from diverse experimental and computational data. Thus, this methodology is recommended for the challenging elucidation of TM protein structures, when single computational and experimental methods are insufficient or not available. Finally, we would like to draw attention to an alternative direction of investigation of protein function when its' 3D structure is not known. When the 3D structure of the protein or at least of close homologs is not available, there are still some methods for the investigation of functions of such proteins based on structure-free approaches such as pharmacophore and structure–activity modeling. The *in silico* ligand-based models designed based on quantitative structure activity relationships (QSAR) are one of very few methods that can be used in the absence of the resolved 3D protein structure. Once the QSAR model is constructed, it can offer valuable information on the structural features of substrates that are of significance for their transport ability (*e.g.* drug absorption/excretion). For example, despite of lack of structural data, such effective QSAR models were also possible to develop for this case study protein BTL [29], [30], [154]. However, this is completely new story for another review.

## Figures and Tables

**Table 1:** The comparative performance of state-of-art freely available predictors for: α-transmembrane regions - based on a benchmark dataset of 38 α-TM proteins [81], [91], β-transmembrane regions - based on a benchmark dataset of 35 β-TM proteins [57].

α-transmembrane region predictors					β-transmembrane region predictors				
Program	SE	PR	Method	Reference	Program	SE	PR	Method	Reference
DAS-TMfilter	79	76	HA & DAS	Cserzo et al., 2004 [107]	B2TMpred	83	42	NN	Jacoboni et al., 2001 [82]
HMMTOP	90	89	HMM	Tusnády & Simon, 2001 [76]	ConBBPred	56	86	consensus	Bagos et al., 2005 [75]
MemBrain	83	87	NN	Shen & Chou, 2008 [108]	PredβTM	84	73	SVM & NN	Roy Choudhury et al., 2015 [57]
MEMSAT3	91	82	NN	Jones, 2007 [71]	TBBpred	74	41	SVM & NN	Natt et al., 2004 [98]
OCTOPUS	90	89	NN	Viklund & Elofsson, 2008 [95]	TMBETA-NET	72	46	NN	Gromiha et al., 2005 [102]
Philius	92	89	DBN	Reynolds et al., 2008 [78]	TMBpro	75	70	NN	Randall et al., 2008 [84]
Phobius	91	86	HMM	Käll et al., 2004 [93]					
PredαTM	92	90	SVM & NN	Roy Choudhury et al., 2013 [91], [103]					
PRED-TMR	92	82	HA	Pasquier et al., 1999 [64]					
SCAMPI	90	89	HMM	Bernsel et al., 2008 [96]					
SOSUI	88	80	HA	Hirokawa et al., 1998 [65]					
SVMtm	90	80	SVM	Yuan et al., 2004 [109]					
SVMtop	90	89	SVM	Lo et al., 2008 [110]					
TMHMM	91	87	HMM	Krogh et al., 2001 [79]					
TMpred	87	87	SA	Hofmann & Stoffel, 1993 [73]					
TOPCONS	90	90	consensus	Bernsel et al., 2009 [80]					
TopPred II	86	88	HA	Claros & von Heijne, 1994 [67]					

HA - hydropathy analysis, SA – statistical analysis, DAS - Dense Alignment Surface, DBN - Dynamic Bayesian network.

SE (sensitivity) - % of all observed TM regions predicted correctly.

PR (precision) - % of all TM regions that are correctly predicted.
